# Dynamics of ADH and related genes responsible for the transformation of C_6_‐aldehydes to C_6_‐alcohols during the postharvest process of oolong tea

**DOI:** 10.1002/fsn3.1272

**Published:** 2019-11-25

**Authors:** Zi‐Wei Zhou, Qing‐Yang Wu, Zhi‐Ling Yao, Hui‐Li Deng, Bin‐Bin Liu, Chuan Yue, Ting‐Ting Deng, Zhong‐Xiong Lai, Yun Sun

**Affiliations:** ^1^ Key Laboratory of Tea Science in Fujian Province College of Horticulture Fujian Agriculture and Forestry University Fuzhou China; ^2^ Institute of Horticultural Biotechnology Fujian Agriculture and Forestry University Fuzhou China

**Keywords:** alcohol dehydrogenase, *Camellia sinensis*, turnover, volatile C_6_‐compounds

## Abstract

Aroma is an important index of tea quality. The volatile C_6_‐compounds formed from linoleic and linolenic acids in tea leaf lipids are essential components of tea. C_6_‐compounds are formed and transformed during the postharvest process of tea leaves. However, the metabolic flux of these C_6_‐compounds, the activities of related enzymes, and the transcription of related genes during the postharvest process of oolong tea remain unclear. In this study, the chemical profiles of C_6_‐aldehydes and C_6_‐alcohols, the pattern of ADH enzyme activity, and the level of *CsADH* gene expression during the postharvest process of oolong tea were investigated. We found that the turnover process had a positive effect on the accumulation of C_6_‐alcohols and simultaneously induced ADH activity, especially during the withering stage. The expression of *CsADH* peaked during the turnover stage. The relative expression level of CSA019598 typically increased during the postharvest process. Correlation analysis demonstrated that CSA019598 expression increased as ADH activity increased. This finding suggests that CSA019598 may play a prominent role in regulating ADH. These results advance our understanding of C_6_‐compound formation during the postharvest process of oolong tea. We aim to evaluate how green leaf volatiles affect the enzymatic formation and genetic transcription of aromatic compounds in oolong tea in future studies.

## Graphical abstract

(a) ADH reduces C6‐Aldehydes into C6‐alcohols during the postharvest process of oolong tea under mechanical wounding conditions. (b) CSA019598 screened from tea plant genome might be an available CsADH gene which plays a major regulatory role during the postharvest process of oolong tea.

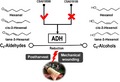

## 1 INTRODUCTION

Tea is the second most popular beverage in the world after water. As one of the six Chinese traditional tea categories, semifermented oolong tea is favored by consumers because of its natural floral and fruity aroma (Ma et al., 2018; Wang, Meckling, & Marcone, 2017). Previous research has shown that the aroma formation in oolong tea mainly depends on the preharvest and postharvest treatment of fresh tea leaves, including light, insect, steaming, firing, heat‐rolling, withering, solar‐drying, and fermentation (Kawakami & Kobayashi, 2001; Mei et al., 2017; Xu et al., 2018; Zeng et al., 2016). According to their metabolic origin, tea volatile compounds can be divided into four major classes: fatty acid derivatives, phenylpropanoids/benzenoids, terpenoids, and norisoprenoids (Fu et al., 2017; Yang, Baldermann, & Watanabe, 2013). Among these classes, fatty acid derivatives play a vital role determining the tea aroma during the postharvest process.

**Figure 1:**
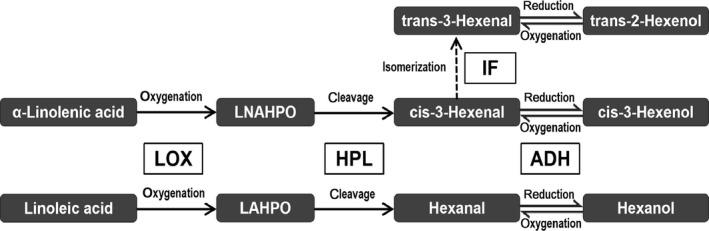
LOX‐HPL pathway for volatile C_6_‐compounds derived from unsaturated fatty acids. LOX: lipoxygenase, http://www.chem.qmul.ac.uk/iubmb/enzyme/EC1/13/11/12.html; HPL: hydroperoxide lyase, http://www.chem.qmul.ac.uk/iubmb/enzyme/EC4/1/2/.html‐; ADH: alcohol dehydrogenase, http://www.chem.qmul.ac.uk/iubmb/enzyme/EC1/1/1/1.html; IF: isomerization factors; LNAHPO: hydroperoxide of α‐linolenic acid; LAHPO: hydroperoxide of linoleic acid

An important cash crop, the tea plant encounters light, heat, and wounding stresses during postharvest process (Cho et al., 2007; Gui et al., 2015). C_6_‐compounds, including hexanal, cis‐3‐hexenal, hexanol, and cis‐3‐hexenol, are volatiles derived from long‐chain unsaturated fatty acids (e.g., linolenic acid and linoleic acid). The three enzymes LOX (lipoxygenase, http://www.chem.qmul.ac.uk/iubmb/enzyme/EC1/13/11/12.html), HPL (hydroperoxide lyase, http://www.chem.qmul.ac.uk/iubmb/enzyme/EC4/1/2/.html.‐), ADH (alcohol dehydrogenase, http://www.chem.qmul.ac.uk/iubmb/enzyme/EC1/1/1/1.html), and an isomerization factor in the LOX‐HPL pathway are responsible for the biosynthesis of the volatile C_6_‐compounds in tea leaves, as shown in Figure 1 (Hatanaka & Harada, 1973; Hatanaka, Kajiwara, & Sekiya, 1976; Kajiwara, Harada, & Hatanaka, 1975; Matsui et al., 2000; Qian, Sun, Xu, Zhu, & Xu, 2016; Sekiya, Kawasaki, Kajiwara, & Hatanaka, 1975; Sekiya, Numa, Kajiwara, & Hatanaka, 1976). Among them, ADH, a zinc‐containing enzyme involved in short‐chain alcohol metabolism of higher plants, is activated under biotic and abiotic stress (Heinstra, Geer, Seykens, & Langevin, 1989; Kagi & Vallee, 1960; Mangolin, Prioli, & Machado, 1994). Following ADH activation, C_6_‐aldehydes are reduced to C_6_‐alcohols under the oxygen deficit condition through the enzymatic action of ADH, regarded as an indicator of anaerobic conditions (Francis et al., 1974; Sekiya, Kajiwara, & Hatanaka, 1984). C_6_‐aldehydes and C_6_‐alcohols are key components of green leaf volatiles (GLVs), which are not only plant‐defense compounds but also important precursors of tea aroma (Groot et al., 2008; Mu et al., 2014; Ono et al., 2016). Hence, the activity of ADH and the expression of its related genes are fundamental to the development of tea quality during postharvest process.

A previous study on ADH and its related genes focused on the stress response of plants over a long time period. Much work has shown that *ADH* genes can respond to dehydration (Cirilli et al., 2012), low temperature (Strommer, 2011), and abscisic acid (Lu, Paul, Mccarty, & Ferl, 2018 ^) ^and play essential roles in fruit ripening (Hao, Cao, Jin, Tang, & Qi, 2016), seedling development (Díaz, Fernández, & Martínez, 2016), and pollen formation (Freeling, 1978). In the field study of tea plants, Xin, Sun, and Chen (2013) revealed the expression patterns of the *CsADH1* gene under tea geometrid feeding, mechanical wounding, and treatment with jasmonic acid and salicylic acid. Peng (2015) studied the expression patterns of the ADH gene in the leaves of *Camellia sinensis cv*. Longjing 43 under stress from different insects. Sekiya *et al*. (Francis et al., 1974) studied the seasonal variation of ADH in tea leaves. However, a little work has been performed on the relationship between ADH and the development of tea quality during the postharvest process.

In the present study, one bud and three or four leaves of the tea plant (*Camellia sinensis* cv. Huangdan) were sampled during the postharvest process of oolong tea. The chemical profiles of the C_6_‐aldehydes and C_6_‐alcohols, the activity of the enzyme ADH, and the expression of *CsADH* (accession number: HM440157.1) during the postharvest process of oolong tea were investigated. Furthermore, potential *CsADH* genes were selected and analyzed using the tea tree genome database. Our study provides a reference for researches into the changes of volatile C_6_‐compounds during the postharvest process of oolong tea. In addition, the results of this study provide insight into the mechanisms underlying aroma formation during the manufacturing of oolong tea.

## 2 MATERIALS AND METHODS

### 2.1 Plant material and postharvest treatments

Tea leaves were collected from *Camellia sinensis* var. Huangdan, a popular cultivar in the oolong tea production area. Tea leaves were sampled in the university research field of Fujian Agriculture and Forestry University (26°04'N, 119°14'E) (Fuzhou, China) from 3 to 5 p.m. on 1st October 2018 under sunny conditions. The fresh leaves (P) were sampled at 4.00 p.m.; the solar withering (SW) treatment spanned 30 min. Turnover treatment is an important step in the postharvest process of oolong tea. Turnover was carried out three times, and the turnover machine maintained a rate of 25 rounds per min. Tea leaves were turned over for five min each time, and then, indoor withering treatment was performed for 1.0 hr. Leaves at time points designated 1T, 2T, and 3T were sampled after the first, second, and third stage, respectively, of turnover treatment and composed the experimental group (EG), with the weight of leaves sampled at each time point totaling 500 g. The remaining indoor withered leaves were used as a control group (CK) and were not subjected to turnover treatment; subgroups CK1 to CK3 were sampled at the same time points as the EG leaves, with 500 g of leaves sampled at each time point. Sampling for each treatment was repeated three times. All samples were wrapped in tin foil, rapidly fixed with liquid nitrogen, and then placed in an ultra‐low temperature freezer (−70°C).

### 2.2 Detection of volatile components C_6_‐aldehydes and C_6_‐alcohols

Each sample was subjected to freeze‐drying as pretreatment, and 2.0 g (accurate to 0.001 g) of milled sample was added to 5.0 ml of ddH_2_O for testing. A GC680+SQ8T+HS40 GC/MS (Perkin Elmer) and headspace gas chromatograph with a VF‐5MS capillary column (Palo Alto, USA, 30 m × 0.25 mm × 0.25 μm) was used for detection. The sample handling conditions were as follows: heating temperature of headspace bottle: 80°C, sampling needle temperature: 100°C, equilibrium temperature of sample bottle: 150°C, sample equilibration time: 5 min, handling time: 1.0 min, pressurization time: 2.0 min, needle extraction time: 0.5 min. The capillary column was a VF‐5MS. The chromatographic separation conditions were as follows: sample inlet temperature: 250°C, heating program: initial temperature 40°C for 5 min, increase at 2°C/min to 70°C, then increase at 10°C/min to 230°C. The mass spectrometric detection conditions were as follows: mass spectrometry interface temperature: 250°C, transmission line temperature: 120°C, ion mode: EI source, ion source temperature: 200°C. The obtained mass spectral data were qualitatively analyzed in the NIST library.

### 2.3 Detection of ADH enzyme activity

The detection of ADH activity was carried out following the instructions provided with the ADH activity assay kit (Keming Biotechnology Co., Ltd). The activity was calculated according to the liquid volume. The formula is as follows:

$$\text{ADH}\;\left(\mu \text{mol}\;\min^{-1}\;{\text{ml}}^{-1}\right)\;=\;\left[\left(\Delta A\;\text{measuring}\;\text{tube}-\Delta A\;\text{blank}\;\text{tube}\right)÷\varepsilon ÷d\times V_{\mathrm{tc}}\times 106\right]÷V_{\text{sample}}÷T\;=\;1.61\;\times \;\left(\Delta A\;\text{measuring}\;\text{tube}-\Delta A\;\text{blank}\;\text{tube}\right)$$

*Note*: Activity unit definition: enzyme oxidizes 1 μmol of NADH per minute per milliliter of substrate at 25°C, NADH for each enzyme unit.

ε: NADH molar extinction coefficient, 6.22 × 103 L/mol cm^−1^; d: cuvette light path, 1 cm; Vtc: total volume of reaction system, 1,000 µl = 0.001 L; Cpr: protein concentration in supernatant, mg/ml; W: sample quality, g; Vsample: volume of the supernatant added to the reaction system, 100 µl = 0.1 ml; T: reaction time, 1 min.

### 2.4 Screening conditions for the genome database and bioinformatics analysis tool

Based on the tea plant whole genome (CSA) database (Xia et al., 2017), a search was performed of the published functional annotations (TSV) based on the accession number of the typical protein domain of ADH. In the Pfam TSV, the accession numbers (PF08240, PF00107, PF01408) of the three typical functional domains of ADH were searched as keywords. The simple modular architecture research tool (SMART, http://smart.emblheidelberg.de) (Letunic, Doerks, & Bork, 2009) was used to analyze the ADH amino acid sequence. The Basic Local Alignment Search Tool (BLAST, (https://blast.ncbi.nlm.nih.gov/Blast.cgi) Altschul & Koonin, 1998) was use to download homologous *CsADH* gene sequences. Molecular Evolutionary Genetics Analysis version 7.0 (MEGA 7.0) (Sudhir, Glen, Daniel, & Koichiro, 2012) and Interactive Tree of Life (iTOL, (https://itol.embl.de/upload.cgi) Ivica & Peer, 2016) were used to built and edit, respectively, a phylogenetic tree. A motif search of the ADH gene was carried out on the Internet (http://www.genome.jp/tools/motif/) (Kim et al., 2015).

### 2.5 Semiquantitative RT‐PCR and Real‐time quantitative PCR assay

Total RNA was extracted in accordance with the instructions provided with the RNAprep Pure Plant Kit (Tiangen). An ultra‐micro nucleic acid analyzer (Thermo Scientific) and DYY‐6B electrophoresis apparatus (Beijing Liuyi Instrument Factory) were used to detect the completeness and concentration of the total RNA extracted. A PrimeScript^TM^ RT Reagent Kit with gDNA Eraser Kit (TaKaRa) was used to synthesize the cDNA. Semiquantitative RT‐PCR assay was performed to measure the expression levels of ADH genes using a C1000 Touch Thermal Cycler (Bio‐Rad). *CsEF‐1α *(Fu et al., 2017) was used as a reference gene. The reaction volume for PCR amplification was 25 μl and comprised 12.5 μl of TransStart FastPfu DNA polymerase, 9.5 μl of ddH_2_O, 1 μl of 3× diluted template cDNA (400 ng μl^−1^), and 1 μl of each primer (10 nmol ml^−1^). PCR was performed under the following conditions: 3 min at 94°C for denaturation; 35 cycles of 30 s at 94°C (denaturation), 30 s at 52°C (annealing), and 90 s at 72°C (extension), and a final step of 10 min at 72°C for extension. The electrophoresis method was used to detect DNA bands of candidate reference genes from the PCR products with two percent agarose gel.

The RT‐qPCR primers for the tea plant ADH‐related genes were designed using DNAMAN 6.0 (Table S1), and the expression levels of *CsADH* genes during the postharvest process of oolong tea were detected using a LightCycler® 480 instrument according to the instructions provided with the Takara SYBR Premix Ex Taq^TM^ kit. Three biological determinations were repeated three times using the relative quantification method 2^−△△Ct^ (Livak & Schmittgen, 2001). The expression levels of the ADH‐related genes were normalized by *CsEF‐1α* expression (Fu et al., 2017). The reaction system was performed in a volume of 20 μl comprising 1.0 μl of cDNA, 10 μl of SYBR II, 0.8 μl of up‐ and downstream primers, and 7.4 μl of ddH_2_O. PCR was performed following the same program used for the semiquantitative RT‐PCR assay.

### 2.6 Data analysis

PASW Statistics 18.0 software was used for analysis, and differences in ADH activity and the relative expression of related genes were analyzed using the least significant difference test (LSD) (Thiele‐Bruhn & Beck, 2005) at a significance level of *p* < .05. Differences between the experimental group and the control group were evaluated at significance levels of *p* < .05 and *p* < .01. Correlations of ADH activity and the relative expression levels of related genes in the experimental and control groups were analyzed using Spearman correlation analysis.

## 3 RESULTS

### 3.1 Total contents and compositions of C_6_‐aldehydes and C_6_‐alcohols during the postharvest process of oolong tea

**Figure 2:**
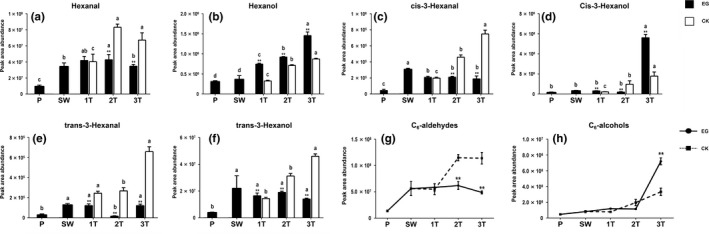
Changes in C_6_‐aldehyde and C_6_‐alcohol levels during postharvest process of oolong tea. Samples were obtained at each stage of the manufacturing process (P, fresh leaves; SW, solar withering; 1T, the first turnover (and control); 2T, the second turnover (and control); 3T, the third turnover (and control)). The relative amounts of six kinds of C6‐compounds, hexenal (a), hexanol (b), cis‐3‐hexenal (c), cis‐3‐ hexenol (d), cis‐3‐hexenal (e), and trans‐2‐hexenol (f), were detected. The total amounts of C_6_‐aldehydes (g) and C_6_‐alcohols (h) were detected. Note: One or two asterisks denote a statistically significant difference (*p < .05; **p < .01) in relative amount between the EG group and the CK group at the same sample time. Values within the same stage followed by the same letter do not differ significantly (*p* > .05). Error bars indicate the standard error (*SE*) of the mean

The total contents of C_6_‐aldehydes and C_6_‐alcohols during the postharvest process of oolong tea were detected via GC‐MS. C6‐aldehydes, including hexanal, cis‐3‐hexenal and trans‐3‐hexenal, were detected. The relative amounts of hexanal and trans‐3‐hexenal exhibited a decreasing trend in the EG, whereas the relative amount of cis‐3‐hexenal increased slightly but not significantly. The relative amounts of the three kinds of C_6_‐aldehyde all showed increasing trends, and the difference between the EG and CK group reached significance at both the 2T and 3T stages. C_6_‐alcohols, including hexanol, cis‐3‐hexenol, and trans‐3‐hexenol, were also detected; their levels continuously increased after intermittent mechanical wounding at turnover in the EG, reaching a peak at 3T. Although the relative amounts of the four alcohols also increased in the CK group, the differences from EG were generally significant (*p* < .05 or *p* < .01). These results indicated that the turnover process of oolong tea can significantly transform C_6_‐aldehydes into C_6_‐alcohols. This indicates that although some C_6_‐aldehydes with unpleasant GLVs evaporated, others were converted into soft and fragrant C_6_‐alcohols (Figure 2a–f). Furthermore, the total contents of C_6_‐aldehydes and C_6_‐alcohols at the SW stage were increased by turnover. Turnover treatment slowed the accumulation of C_6_‐aldehydes. The accumulation was further slowed during the 2T and 3T stages. A large amount of C_6_‐aldehydes accumulated in the CK group, and there were extremely significant differences between the EG and the CK group at the 2T and 3T stages (Figure 2h). In contrast, the total amount of C_6_‐alcohols was higher in the EG than in the CK group, and there was an extremely significant difference between 3T and CK3 (Figure 2g).

### 3.2 Changes in ADH activity during the postharvest process of oolong tea

**Figure 3:**
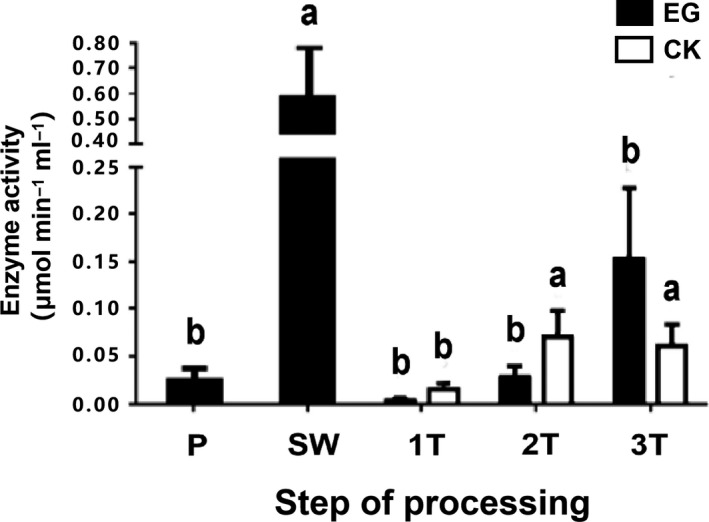
Analysis of ADH activity during the postharvest process of oolong tea. Samples were obtained at each stage of the manufacturing process (P, fresh leaves; SW, solar withering; 1T, the first turnover (and control); 2T, the second turnover (and control); 3T, the third turnover (and control)). Note: One or two asterisks denote a statistically significant difference (*p < .05; **p < .01) between the EG group (black bars) and the CK group (white bars) in activity within a sample time. Values within the same stage followed by the same letter do not differ significantly (*p* > .05). Error bars indicate the standard error (*SE*) of the mean

The analysis result of ADH activity measured by visible light spectrophotometry showed that ADH activity was low in P (0.020 U_2_) but then increased significantly, peaking in the SW phase (0.550 U_2_). At the beginning of the turnover stage, the activity of ADH was significantly lower than that during SW. ADH activity was lowest during the 1T stage (0.003 U_2_), as the mechanical wounding due to turnover inhibited the activity of ADH in solar‐withered leaves. However, as turnover progressed, ADH activity gradually increased. The ADH activity of CK1 and CK2 was higher than that of EG until the third turnover, at which time the enzyme activity of 3T was higher than that of CK3 (Figure 3).

### 3.3 Screening and sequence analysis of ADH genes based on the tea plant genome

**Figure 4:**
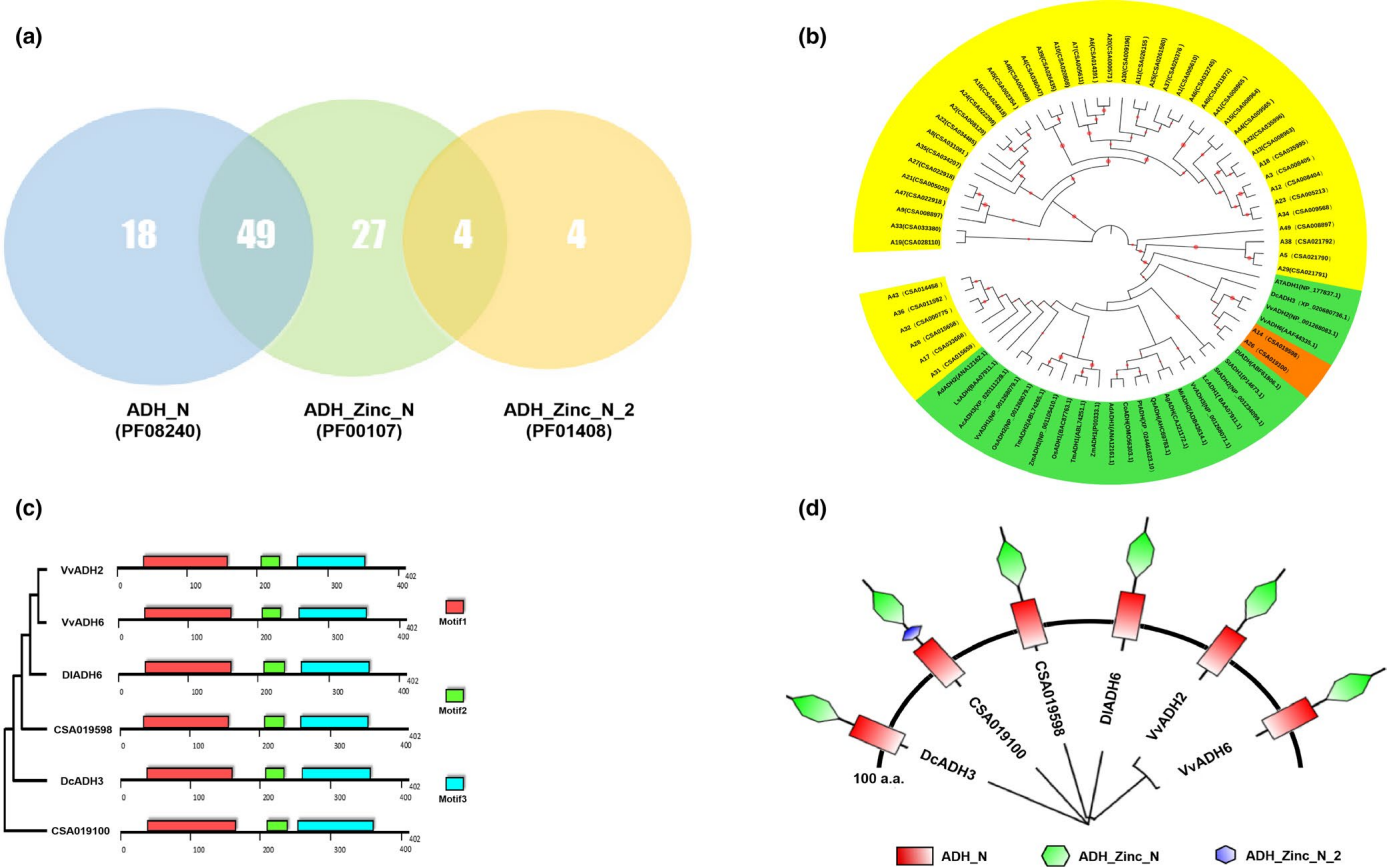
The selection and analysis of ADH genes in a tea plant (CSA) genome database. (a) Venn diagram of the number of genes; 3 AHD (AHD_N(blue), AHD_Zinc_N(green), and AHD_Zinc_N_2(yellow)), typical functional domains of the tea tree. 102 ADH candidate genes were selected. There were 49 ADH genes with both ADH_N and ADH_N_2 domains and only 4 ADH genes with both ADH_N_2 and ADH_N_2_N functional domains. (b) Phylogenetic tree of 49 ADH genes (yellow and orange) in the tea plant and 25 ADH genes (green) in 17 other species. The red circles indicate the bootstrap values of the branches; the larger the circle area, the larger the bootstrap value. AdADH1, AdADH2: kiwi fruit; LsADH: lettuce; CoADH: jute; AgADH: European alder; AtADH1: Arabidopsis thaliana; AcADH3: pineapple; MiADH: mango; PtADH: poplar; QsADH: Quercus suber; OsADH1, OsADH2: rice; TmADH1, TmADH2: wheat; StADH1, StADH2: potato; ZmADH: maize; DcADH: Dendrobium officinale; DlADH: longan; LcADH1: VvADH1, VvADH2, VvADH6, VvADH3: grape. (c) Motif analysis of ADH amino acid sequences. Red, green, and blue boxes represent Motif1 (124 a.a.), Motif2 (30 a.a.), and Motif3 (97 a.a.), respectively. (d) Domain analysis of ADH amino acid sequences. The red boxes, green hexagons, and blue hexagon represent the ADN_N domain, ADN_Zinc_N domain, and ADN_Zinc_N_2 domain, respectively

A total of 102 candidate fragments were obtained based on the CSA database, including 67 fragments of ADH_N, 80 fragments of ADH_zinc_N, 8 fragments of ADH_zinc_N_2, and 6 fragments of Rossmanfold. The largest intersection where the ADH_N and ADH_zinc_N groups align contained 49 fragments (Figure 4a), which are labeled A1–A49 in Table S2.

Multiple sequence alignment and phylogeny evolution analysis were carried out on the 49 selected segments, and 25 *ADH* genes of 17 species were found and distributed across two parts of the phylogenetic tree. One part (colored yellow in the figure) represents the 49 CDS fragments screened from the CSA database, and the other part (colored green in the figure) represents the ADH genes from other species registered in GenBank (Figure 4b). It is noteworthy that A14 and A26 exhibit short genetic distances from the ADH genes of grape, longan, and *Dendrobium candidum*. These two CDSs were designated CSA019598 and CSA019100 in the CSA database and belong to the ADH2 family of genes in plants. Therefore, CSA019598 and CSA019100 were regarded as the main target genes in our subsequent analysis. The two segments were subjected to clustering analysis using the neighbor‐joining (NJ) method. Compared with CSA019100, CSA019598 has a closer genetic distance to *VvADH2, VvADH6* of grape, and *DlADH2* of longan. CSA019598 had high sequence similarity to *VvADH2* and *VvADH6*, with values of 87.37% and 87.89%, respectively. The amino acid sequences of CSA019598 and CSA019100 were compared with the conserved domains from other species with close genetic relationships with tea tree. The results showed that the amino acid sequence of the ADH gene of the tea plant was highly consistent with that of grape, *Dendrobium*, and longan, indicating that the amino acid sequence of *ADH* is highly conserved in plants. In the 234–256 locus region of the CSA019100 fragment, there was a redundant series of 23 amino acids, which was regarded as an interval sequence of CSA019100 (Figure S1). The deletion of this fragment may be a transcript produced by alternative splicing of the gene, providing a target site for posttranscriptional modification. The amino acid sequences of CSA019598 and CSA019100 were subjected to conserved element analysis with other species using the MOTIF Search online software. The results showed that the *ADH* gene segments of the tea plant, grape, longan, and *Dendrobium* all possessed three highly conserved motifs, indicating that the fragments screened from the CSA database were consistent both in location and in length with the motifs of ADH genes in other plants. The defined Motif1, Motif2, and Motif3 were in the ranges of 20–140 a.a., 205–235 a.a, and 245–320 a.a., respectively (Figure 4c). The redundant segments of CSA019100 separated Motif2 and Motif3. Furthermore, we found two main domains, AHD_N and AHD_Zinc_N, in these amino acid sequences (Figure 4d). Motif1 belongs to the ADH_N domain, and Motif 2 and Motif 3 belong to the ADH_zinc_N domain.

### 3.4 Semiquantitative RT‐PCR and RT‐qPCR analysis of ADH‐related genes

**Figure 5:**
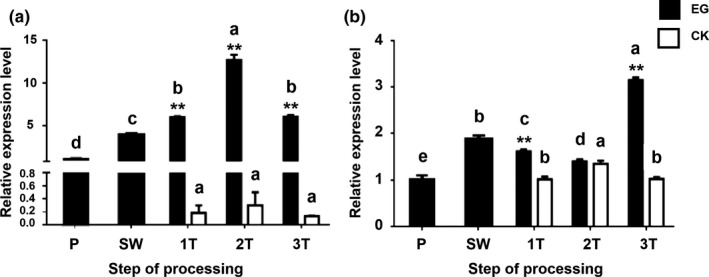
Relative expression of the *CsADH* gene (a) and CDS of CSA019598 (b) during the postharvest process of oolong tea. Samples were obtained at each stage of the manufacturing process (P, fresh leaves; SW, solar withering; 1T, the first turnover (and control); 2T, the second turnover (and control); 3T, the third turnover (and control)). a, relative expression level of the *CsADH* gene (GenBank accession: HM440157.1); b, relative expression level of CDS of CSA019598. Note: One or two asterisks denote a statistically significant difference (*p < .05; **p < .01) between the EG group (black bars) and the CK group (white bars) in relative expression at the same sample time. Values from the same vessel followed by the same letter do not differ significantly (*p* > .05). Error bars indicate the standard error (*SE*) of the mean

RT‐qPCR assay showed that the relative expression level of *CsADH* increased significantly from the P stage to the 2T stage and then began to decrease in 3T. In the CK group, the variation in ADH activity was consistent with changes in ADH transcript level, but the difference in ADH activity between the CK group and EG was not significant. The relative expression levels of the *CsADH* gene were significantly different between the EG and the CK group (*p* < .05; Figure 5a).

Using *CsEF‐1α* as a reference gene, semiquantitative RT‐PCR analysis was performed on CSA019598 and CSA019100. A redundant interval was found in the amino acid sequence analysis of CSA019100, as previously discussed, and the random primer (A26‐1) of the CSA019100 segment and the specific primer of redundant (A26‐2) interval were designed separately in semiquantitation. As shown in Figure S2, compared with the band of the reference gene, the CSA019598 band was bright, whereas CSA019100 did not show a clear band with the different pairs of primers. This result suggests that the expression of CSA019100 may be inhibited during the postharvest process of oolong tea, possibly due to the presence of the redundant sequence. Through qualitative analysis of the semiquantitative results, CSA019100 was excluded. Thus, the subsequent real‐time fluorescence quantitative test focused on CSA019598.

RT‐qPCR assay was performed on the remaining segment, CSA019598. The results showed that the relative expression level of CSA019598 was upregulated during SW, began to decrease after 1T and 2T, and then peaked at 3T. The expression level at each stage differed significantly (*p* < .05) between EG and the CK group. The difference between the first and third turnover was significant. The expression in the CK group initially increased and then decreased, showing an inverted‐V trend, which was consistent with the variation in ADH activity and *CsADH* expression in the CK group during the postharvest process of oolong tea (Figure 5b).

### 3.5 Correlation analysis of ADH activity and the relative expression levels of related genes during the postharvest process

Correlation analysis of the RT‐qPCR results and enzyme activity was performed using SPSS 18.0 software. The correlation between ADH activity and expression of each of *CsADH* and CSA019598 was evaluated. The Spearman correlation coefficients for ADH activity and CsADH gene expression were −0.8082 and 0.420 for the EG and CK group, respectively, and were not significant. In contrast, the coefficients of the correlation between ADH activity and the relative expression of CSA019598 were 0.521 and 0.650 in the EG and the CK group, respectively, and were significant (*p* < .05) and extremely significant (*p* < .01), respectively (Table 1).

**Table 1:** Correlations between ADH activity and the relative expression of related genes

		Relative expression of the *CsADH* gene		Relative expression of CSA019598	
Activity of ADH	Experimental group	R	P	R	P
	Control group	0.420	NS	0.650	**

Note

R: Spearman correlation coefficient. *p < .05, **p < .01, NS = nonsignificant.

## 4 DISCUSSION

### 4.1 Conversion of C_6_‐aldehyde and C_6_‐alcohol compounds by ADH in the postharvest process of oolong tea

As a key enzyme in the LOX pathway, ADH plays important roles in the transformation and formation of volatile C_6‐_compounds during the postharvest process of oolong tea (Heinstra et al., 1989; Matsui et al., 2000; Sekiya et al., 1976). We found that ADH activity peaked during the SW stage. This might have been due to the damage to the fresh tea leaves by harvesting and the abiotic stresses that occurred before the SW treatment was completed. The fresh leaves were exposed to environmental stresses (e.g., light, heat) in vitro (You et al., 2018), leading to an increase of ADH activity. As a key contributor to oolong tea aroma quality (Liu, Yin, Chen, Wang, & Xu, 2018), turnover is a stress that combines water deficit and mechanical wounding; mechanical wounding could be regarded as a novel stress that further damaged the solar‐withered leaves (Zeng, Watanabe, & Yang, 2018). ADH activity plummeted at the beginning stage of turnover treatment, possibly because the leaves were adjusting to a new stress factor. Subsequently, ADH activity gradually increased throughout the turnover process, suggesting that ADH activity responded to the mechanical wounding in vitro. Gui *et al*. (Hatanaka & Harada, 1973) (2015) demonstrated that the activity of key rate‐limiting enzyme in the fatty acid metabolism pathway during the manufacturing process (LOX) gradually increased as turnover proceeded. The present research showed that although the increase in ADH activity, as a downstream enzyme, during turnover was not as extensive as that observed for LOX and HPL, the pattern was similar. The mechanical stress of turnover causes strong frictional collisions among the tea leaves. Breakage of the leaf edge causes enzymatic oxidation of phenolic and other substances of tea leaves, which leads to the conversion and production of secondary metabolites (Jin, Guo, Sun, Ji, & Su, 2003). The detection of volatile components in the samples showed that turnover significantly reduced the level of C_6_‐aldehydes and increased the level of C_6_‐alcohols. Sekiya *et al*. found that a reduction in ADH activity was related to the accumulation of C_6_‐aldehydes in leaves during summer (Francis et al., 1974). Turnover promoted the transformation of volatiles of oolong tea from GLVs to soft, flowery and fruity odors. ADH reduced the C_6_‐aldehydes produced by the LOX‐HPL system to yield C_6_‐alcohols, providing rich precursors for the derivatives of leaf alcohol esters with floral and fruity aroma in oolong tea.

### 4.2 Expression and regulation of the ADH gene during the postharvest process of oolong tea

The amounts of CDS segments of *CsADH* and CSA019598 all increased during the withering process, and the differences between the treated and control leaves were large, consistent with the changes in ADH activity. The relative expression of *CsADH* peaked at 2T, with a lower level at 3T. However, CSA019598 levels first decreased and then increased during the postharvest process. The correlation analysis revealed that ADH activity in both the experimental group and the control group was significantly correlated with the expression of CSA019598. M Cirilli *et al*. found that high temperature and dehydration induced the expression of ADH and *VvADH2* during postharvest process of grape berries and that the physiological response of grapes preceded the changes in gene expression (Cirilli et al., 2012). Our study found that the change in expression level of CSA019598 temporally coincided with the change in ADH activity, indicating that the synchronicity of ADH activity and its related genes might be stronger in leaves than in fruit.

## 5 CONCLUSION

**Figure 6:**
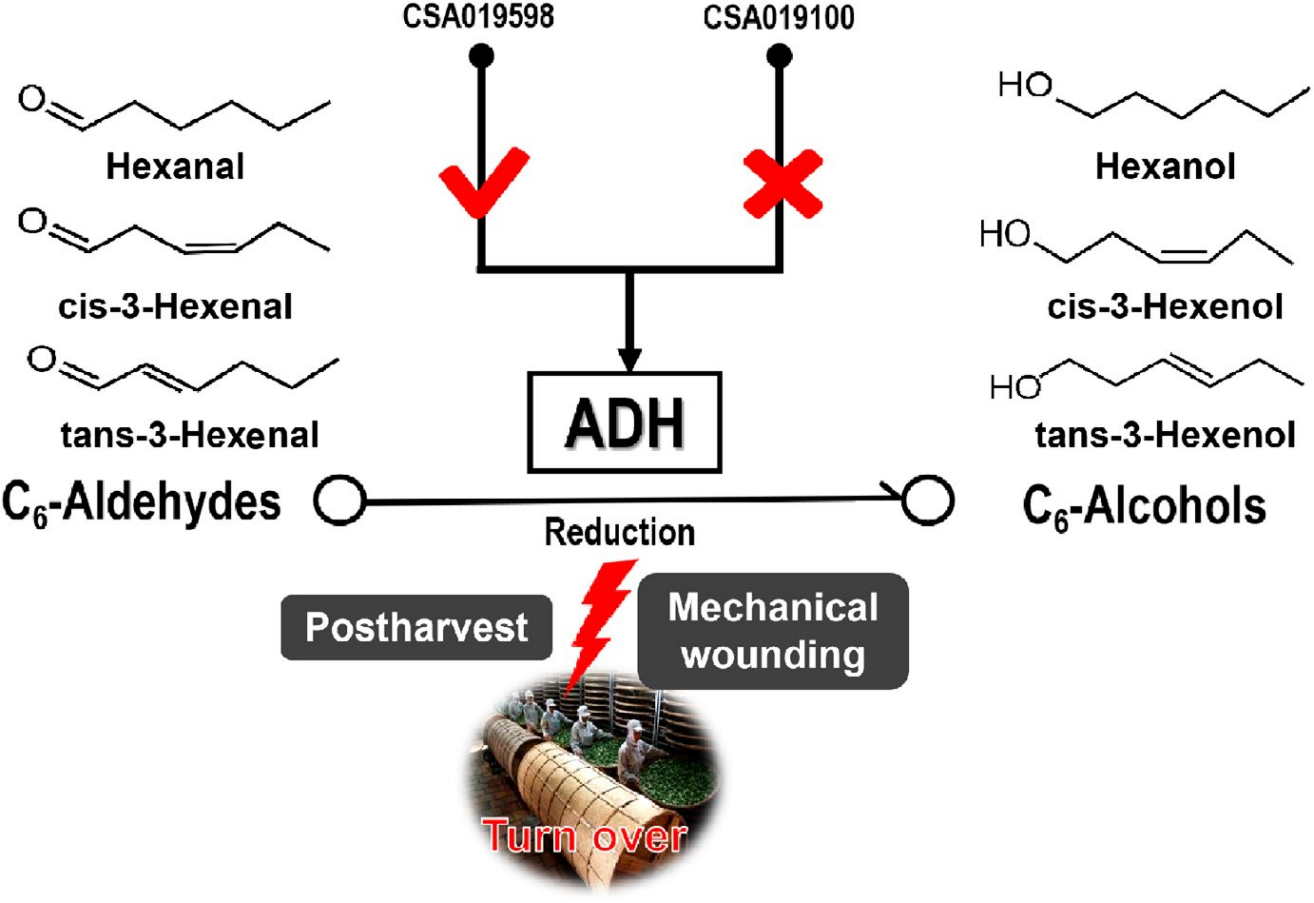
Analysis of the transformation of C_6_‐aldehydes and C_6_‐alcohols under the reduction of ADH during the postharvest process of oolong tea. Tea leaves were sampled at each step of the postharvest process for chemical profiling of C_6_‐aldehydes and C_6_‐alcohols and gene expression profiling. ADH reduces C_6_‐aldehydes (hexanal, cis‐3‐hexenal, and trans‐3‐hexenal) into C_6_‐alcohols (hexanol, cis‐3‐hexenol, and trans‐3‐hexenol) during the postharvest process of oolong tea under mechanical wounding conditions. CSA019598 might be a functional *CsADH* gene that plays a major regulatory role during postharvest process

There may be a regulatory mechanism for the conversion of C_6_‐aldehydes and C_6_‐alcohols in the LOX‐HPL pathway during the postharvest process of oolong tea. Volatile C_6_‐compounds are derived from α‐linolenic and linoleic acid during the postharvest process of oolong tea. Mechanical wounding stimulates ADH activity and changes in the transcription levels of related genes during the turnover stages, and C_6_‐aldehydes, including hexanal, cis‐3‐hexenal, and trans‐3‐hexenal, are gradually reduced to C_6_‐alcohols, including hexanol, cis‐3‐hexenol, and trans‐3‐hexenol. In conclusion, ADH activity and the transcriptional level of *CsADH* genes determine the amounts of volatile C_6_‐alcohols formed, which control the quality of the fresh leaves processed for tea production (Figure 6).

## CONFLICT OF INTEREST

The authors declare that they do not have any conflict of interest.

## ETHICAL REVIEW

This study does not involve any human or animal testing and was approved by the Key Laboratory of Tea Science in Fujian Province, College of Horticulture Fujian Agriculture and Forestry University, and Institute of Horticultural Biotechnology, Fujian Agriculture and Forestry University.

## INFORMED CONSENT

Written informed consent was obtained from all study participants.
