# Supplementary figures and images for: Dynamics of ADH and related genes responsible for the transformation of C6‐aldehydes to C6‐alcohols during the postharvest process of oolong tea
Source: Food Sci Nutr. 2019 Nov 25;8(1):104–13. doi: 10.1002/fsn3.1272 (PMC6977495; doi:10.1002/fsn3.1272)

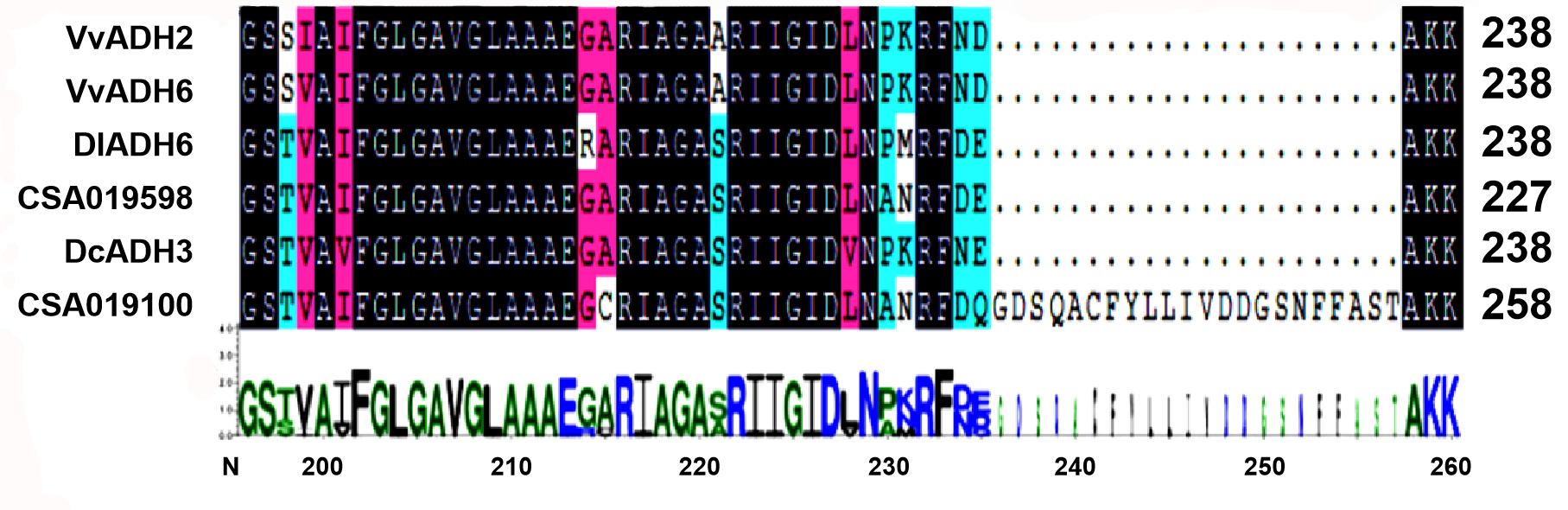

Supplement: Supplementary file 1 [file FSN3-8-104-s001.tiff]

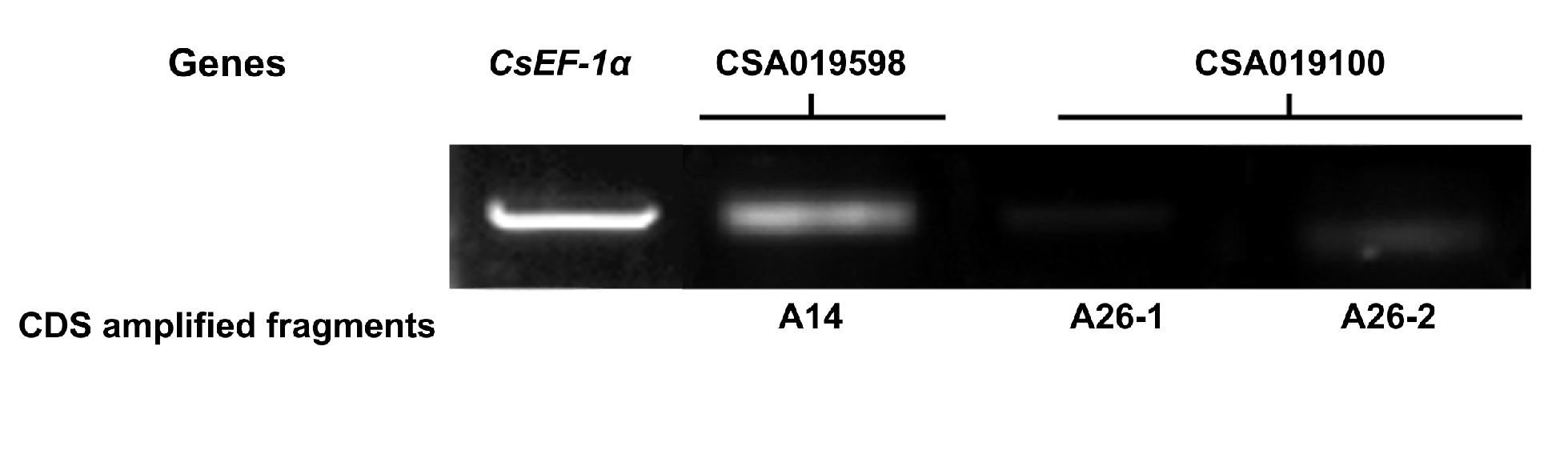

Supplement: Supplementary file 2 [file FSN3-8-104-s002.tiff]
