# Supplementary material for: Dynamics of ADH and related genes responsible for the transformation of C6‐aldehydes to C6‐alcohols during the postharvest process of oolong tea
Source: Food Sci Nutr. 2019 Nov 25;8(1):104–13. doi: 10.1002/fsn3.1272 (PMC6977495; doi:10.1002/fsn3.1272)
Supplement: Supplementary file 3 [file FSN3-8-104-s003.doc]

**Table S1. The specific primers of mRNA for RT-qPCR analysis**

| Gene name | Accession | Base sequence (5’-3’) | Amplification length（bp） | Efficiency value (%) |
| --- | --- | --- | --- | --- |
| *CsADH* | HM440157.1 | F: TGTTGGAAGTGCTGGAACC | 220 | 98.5 |
|  |  | R: CATACCACACACGGCAATG |  |  |
| *CsA14* | CSA019598 | F: CCCTTTGCCACACTGATGT | 107 | 99.0 |
|  |  | R: CTCGCCTACACTTTCCACAAT |  |  |
| *CsA26-1* | CSA019100 | F: GCACCTTCTATGGCAACTACAA | 117 | 100.4 |
|  |  | R: TCTCCGCAAATGTCACTTCA |  |  |
| *CsA26-2* |  | F: ACCAAGGCGATTCTCAAGC | 165 | 104.3 |
|  |  | R:AGATGGAGGAGTGGACCGTA |  |  |
| *CsEF-1α* | KA280301.1 | F: TTCCAAGGATGGGCAGAC | 196 | 97.4 |
|  |  | R: TGGGACGAAGGGGATTTT |  |  |
