# Supplementary material for: Dynamics of ADH and related genes responsible for the transformation of C6‐aldehydes to C6‐alcohols during the postharvest process of oolong tea
Source: Food Sci Nutr. 2019 Nov 25;8(1):104–13. doi: 10.1002/fsn3.1272 (PMC6977495; doi:10.1002/fsn3.1272)
Supplement: Supplementary file 4 [file FSN3-8-104-s004.doc]

**Table S2 The basic information of 49 CDS fragment screened from tea tree genome(CSA)**

| Name | NO. Of Seq. | location | Length (a.a.) | ORF length（bp） |
| --- | --- | --- | --- | --- |
| A1 | CSA005610 | Sc0001753:171158-173953(+) | 361 | 1086 |
| A2 | CSA008129 | Sc0009291:1754-2367(-) | 176 | 531 |
| A3 | CSA008405 | Sc0000520:112691-114262(-) | 329 | 990 |
| A4 | CSA036047 | Sc0001327:16330-18137(+) | 330 | 993 |
| A5 | CSA021790 | Sc0000636:2629-5177(+) | 341 | 1062 |
| A6 | CSA014391 | Sc0000154:327875-330722(+) | 386 | 1161 |
| A7 | CSA005611 | Sc0001753:257816-258671(+) | 215 | 648 |
| A8 | CSA031081 | Sc0000724:530355-536692(-) | 348 | 1047 |
| A9 | CSA029788 | Sc0000002:530642-534990(-) | 440 | 1323 |
| A10 | CSA020808 | Sc0001356:370464-375089(+) | 455 | 1368 |
| A11 | CSA026155 | Sc0000178:51-3495(-) | 360 | 1083 |
| A12 | CSA008404 | Sc0000520:93258-94457(-) | 302 | 909 |
| A13 | CSA008963 | Sc0053268:127602-130160(+) | 360 | 1083 |
| A14 | CSA019598 | Sc0003788:18292-21732(-) | 369 | 1110 |
| A15 | CSA008964 | Sc0053268:132124-133352(+) | 312 | 939 |
| A16 | CSA024818 | Sc0002580:110318-115368(+) | 324 | 975 |
| A17 | CSA033668 | Sc0000174:724871-726686(-) | 348 | 1047 |
| A18 | CSA035995 | Sc0003326:89378-90441(-) | 302 | 909 |
| A19 | CSA028110 | Sc0002305:1838-8718(+) | 377 | 1134 |
| A20 | CSA000573 | Sc0001798:103463-109125(-) | 343 | 1032 |
| A21 | CSA005029 | Sc0002291:40209-41895(-) | 356 | 1071 |
| A22 | CSA034485 | Sc0016175:21-1443(-) | 419 | 1260 |
| A23 | CSA005213 | Sc0004878:65198-66874(-) | 318 | 957 |
| A24 | CSA022299 | Sc0000662:103731-106256(-) | 251 | 756 |
| A25 | CSA026158 | Sc0000178:23756-24990(-) | 279 | 840 |
| A26 | CSA019100 | Sc0001541:47837-51038(-) | 400 | 1203 |
| A27 | CSA022918 | Sc0000008:47416-50137(+) | 254 | 765 |
| A28 | CSA015658 | Sc0000049:1117467-1121247(+) | 400 | 1203 |
| A29 | CSA021791 | Sc0000636:6630-11542(+) | 438 | 1317 |
| A30 | CSA009196 | Sc0054491:59618-63202(-) | 413 | 1242 |
| A31 | CSA015659 | Sc0000049:1129460-1132942(+) | 380 | 1143 |
| A32 | CSA000775 | Sc0000486:241451-244122(+) | 330 | 993 |
| A33 | CSA033380 | Sc0000071:1020341-1022316(-) | 341 | 1026 |
| A34 | CSA009568 | Sc0001072:357115-358134(-) | 299 | 900 |
| A35 | CSA034207 | Sc0001981:311284-316134(+) | 421 | 1266 |
| A36 | CSA011592 | Sc0004350:49627-54210(-) | 391 | 1176 |
| A37 | CSA020376 | Sc0000001:2005401-2010836(+) | 321 | 966 |
| A38 | CSA021792 | Sc0000636:24759-25958(+) | 342 | 1029 |
| A39 | CSA026435 | Sc0000193:34307-38792(+) | 356 | 1071 |
| A40 | CSA011872 | Sc0003013:248484-252466(-) | 297 | 894 |
| A41 | CSA008965 | Sc0053268:138304-139639(+) | 327 | 984 |
| A42 | CSA035996 | Sc0003326:91483-91879(-) | 322 | 969 |
| A43 | CSA014458 | Sc0001951:53177-56539(-) | 380 | 1143 |
| A44 | CSA009565 | Sc0001072:307337-308409(-) | 291 | 876 |
| A45 | CSA002354 | Sc0001825:34746-39707(-) | 349 | 1050 |
| A46 | CSA032745 | Sc0000247:717265-718699(-) | 297 | 894 |
| A47 | CSA022918 | Sc0000008:47416-50137(+) | 364 | 1095 |
| A48 | CSA002499 | Sc0002849:246773-250749(-) | 388 | 1167 |
| A49 | CSA008897 | Sc0000033:233114-251113(-) | 613 | 1842 |
