# Supplementary material for: Dynamics of ADH and related genes responsible for the transformation of C6‐aldehydes to C6‐alcohols during the postharvest process of oolong tea
Source: Food Sci Nutr. 2019 Nov 25;8(1):104–13. doi: 10.1002/fsn3.1272 (PMC6977495; doi:10.1002/fsn3.1272)
Supplement: Supplementary file 5 [file FSN3-8-104-s005.doc]

**Fig. S1. Sequence alignment analysis of CSA019598 and CSA019100 and other species**

Compared with three other species (*Vitis vinifera L., Dimocarpus longan Lour. and Dendrobium nobile Lindl*), CSA019100 had an obvious 22 a.a. length redundant fragment (DGSQACFYLLIVDDGSNFFAST) at the region from 236 to 257.

**Fig. S2. Semi-quantitative RT-PCR of expressed CDS of CSA019598 and CSA019100**

Tea leaves were chosen at the last stage of the manufacturing process, 3T(the third turnover). CSA019598 and CSA019100 were the two CDS numbers filtered from CSA tea tree genome. *CsEF-1α*: the expression of CsACT was used as a reference gene. A14: an amplicon of a pair of random primer of CSA019598, A26-1 and A26-2: two amplicons of two pair of random primers of CSA01900.
